# An Optimized Active Sampling Procedure for Aerobiological DNA Studies

**DOI:** 10.3390/s23052836

**Published:** 2023-03-05

**Authors:** Jyothi Basapathi Raghavendra, Thasshwin Mathanlal, Maria-Paz Zorzano, Javier Martin-Torres

**Affiliations:** 1 Department of Planetary Sciences, School of Geosciences, University of Aberdeen, Aberdeen AB24 3UE, UK; 2 Centro de Astrobiología (CSIC-INTA), Torrejon de Ardoz, 28850 Madrid, Spain; 3 Instituto Andaluz de Ciencias de la Tierra (CSIC-UGR), 18100 Granada, Spain

**Keywords:** bioaerosols, air-filtration, active sampling, commercial off-the shelf (COTS), DNA extraction

## Abstract

The Earth’s atmosphere plays a critical role in transporting and dispersing biological aerosols. Nevertheless, the amount of microbial biomass in suspension in the air is so low that it is extremely difficult to monitor the changes over time in these communities. Real-time genomic studies can provide a sensitive and rapid method for monitoring changes in the composition of bioaerosols. However, the low abundance of deoxyribose nucleic acid (DNA) and proteins in the atmosphere, which is of the order of the contamination produced by operators and instruments, poses a challenge for the sampling process and the analyte extraction. In this study, we designed an optimized, portable, closed bioaerosol sampler based on membrane filters using commercial off-the-shelf components, demonstrating its end-to-end operation. This sampler can operate autonomously outdoors for a prolonged time, capturing ambient bioaerosols and avoiding user contamination. We first performed a comparative analysis in a controlled environment to select the optimal active membrane filter based on its ability to capture and extract DNA. We have designed a bioaerosol chamber for this purpose and tested three commercial DNA extraction kits. The bioaerosol sampler was tested outdoors in a representative environment and run for 24 h at 150 L/min. Our methodology suggests that a 0.22-µm polyether sulfone (PES) membrane filter can recover up to 4 ng of DNA in this period, sufficient for genomic applications. This system, along with the robust extraction protocol, can be automated for continuous environmental monitoring to gain insights into the time evolution of microbial communities within the air.

## 1. Introduction

Biological aerosols are airborne fine solid or liquid particles of biological origin consisting of both living and/or non-living matter. Biological aerosols include bacteria, viruses, fungi, pollen, endotoxins, mycotoxins, organic metabolites, spores, and cell fragments. The study of biological matter in the air is known as “aero microbiology” [1,2,3]. Micron-sized species suspended in the atmosphere can easily be transported on Earth and reach almost any environment, proliferating when the correct nutrient and environmental conditions are met. The study of bioaerosols is expanding, especially concerning microbial ecology research and can provide insights into air-quality monitoring, health concerns, biodefense, atmospheric processes, and meteorology [2,3,4,5,6]. The concentration of bioaerosols is naturally limited in the environment, challenging the sampling process and the subsequent analysis [7,8]. It has been estimated that 37% of the airborne particles constitute aerosols of biological origin, with the average number of bacteria and fungi to be around 1.2 × 10^4^ cells/m^3^ and 7.3 × 10^2^ spores/m^3^, respectively, [9,10] where 65% of the bacteria, fungal cells, and their spores exist as single cells and the rest as agglomerates [11]. This morphological difference in the biological particles has been shown to affect the sampling efficiency [12]. 

The existing sampling methodologies can be classified as passive and active. Passive samplers rely on gravity, natural or artificial electrostatic forces, and turbulent dispersion to allow particles to deposit onto a collection substrate for analysis [13]. Passive sampling can provide only a qualitative analysis as the collected air volume remains unknown. This is mitigated in active sampling, where a powered air-mover samples a known air volume to obtain both quantitative and qualitative analysis. Active sampling is broadly classified into four categories—filtration, impaction, impingement, and electrostatic precipitation. The choice of the sampling technique is determined by the type of analysis to be performed on the collected bioaerosols [14]. In this study, we focus on establishing a protocol to maximize the extraction of quality DNA for genomic studies. These include culture-independent methods, such as molecular techniques and next generation sequencing (NGS) that have led to high-resolution taxonomic profiling [14,15,16,17].

All four active sampling techniques have been successfully used for collecting bioaerosol environmental DNA [18,19]. However, filtration has the highest collection efficiencies (>95%) for particles > 0.5 µm in diameter and is easy to use [20]. Filters can be classified broadly into three categories—fibrous, membrane, and flat based on their morphology. The material, pore size, diameter, and molecular and physicochemical properties of the filter further characterize the choice of filters. Despite their simplicity of use, filtration-based sampling poses a challenge for extracting genetic material from them [8,9,17,21]. The variables of the filter-type, size, and porosity combined with the variables of the sampler—sampling time, rate, and volume play a vital role in determining the quantity and quality of DNA that can be extracted from the filters. The low DNA yield can be mitigated using multiple techniques, such as modified DNA isolation kits, increased sampling time, and flow rate, or pooling of the extracted samples, but this is not always feasible [7,16,22]. Additionally, some sampling procedures can cause stress to the microbes leading to loss of viability during or after collection [7,23]. Increasing the sampling time and sampling rate reduces the relative humidity of the sampling media and has been associated with reduced quality DNA, owing to desiccation of the cell walls affecting the viability [24]. Maintaining an intact cell wall by reducing the stress during sampling is crucial to maximizing the yield of quality DNA. 

Due to the high heterogeneity and unspecified viability of the biological matter in the ambient air, with varying environmental and meteorological conditions at the time of sampling, a robust method that can operate under all conditions is needed. There does not exist a universal sampler for bioaerosols that can meet the demands of high heterogeneity and unspecified viability of the biological matter in the ambient air, and this is a major shortcoming in the field of bioaerosols study. To address this shortcoming, we have designed an optimized, portable, closed bioaerosol sampler based on membrane filters and commercial off-the-shelf (COTS) components, and we demonstrate its successful end-to-end operation. This sampler is designed to operate autonomously outdoors for prolonged times, accumulating ambient bioaerosols in a single filter while avoiding user contamination. The bioaerosol sampler is described in Section 2.1. The design of this aerosol sampler has been constructed to allow for future upgrades to automize the use of independent filters in a sequential manner and capture snapshots of the microbial communities of the bioaerosols in the atmosphere under varying environmental and meteorological conditions. A collection of existing filters was selected for testing. These are described in Section 2.2. Using COTS components, a bioaerosol chamber was developed; in this chamber, we monitored with sensors the environmental conditions of temperature, relative humidity, and atmospheric pressure and tested different filters and DNA extraction protocols against an artificially generated bioaerosol with a fixed bio-load content, as described in Section 2.3. A control preparation for the bioaerosol generation was specifically designed for this experiment (see Section 2.4), and three DNA extraction kits from Qiagen were tested (see Section 2.5). Two experiments were performed in this study. The first experiment was to determine the optimum filter and the standardised protocol to extract a quantifiable amount of genetic material with the highest quality; see Section 3.1. The second experiment was performed to validate the best-performing filters chosen from the first experiment in a representative outdoor environment; see Section 3.2. To illustrate how the morphology of the filter and its physicochemical properties affect the sampling and extraction efficiency, we performed scanning electron microscopy (SEM) of all four filters after sampling with bioaerosol positive controls in the bioaerosol chamber; the results are summarized in Section 3.3.

## 2. Methodology

### 2.1. Bioaerosol Sampler

**Figure 1:**
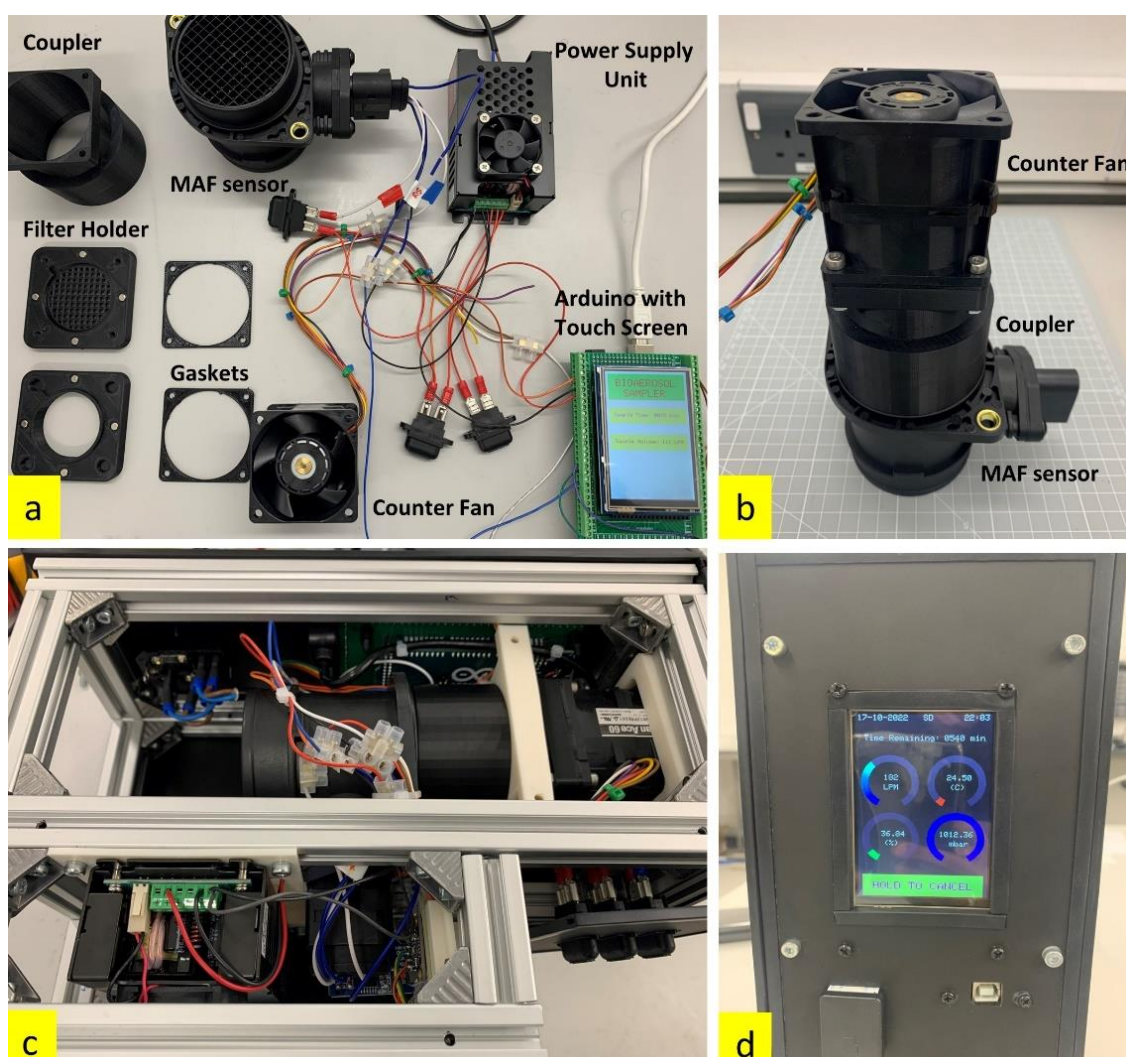
(**a**) Components of the bioaerosol sampler; (**b**) core of the bioaerosol sampler with the counter fan coupled to the MAF sensor; (**c**) assembled components of the bioaerosol sampler inside the housing; (**d**) touch screen GUI showing the parameters of sampling in progress.

Commercial samplers have limitations on the time and flow rate of sampling. Portable filter-based bioaerosol samplers such as the Sartorius MD8 Airport, offer a maximum sampling rate of 125 L/min and are limited to 4 h of sampling time. The L-series portable air sampler from Munro Instruments offers a sampling time of up to 30 h, with the L100 model offering a maximum of 100 L/min sampling rate. Aerobiological investigations need a minimum of 24 h of sampling to obtain enough DNA mass. For this purpose, a portable bioaerosol sampler was built using COTS components, see Figure 1. It was constructed using a counter-rotating fan (San Ace60 9CRA0612P6K001) that operates at 12 V DC with a maximum airflow of 2.28 m^3^/min and a static pressure of 1130 Pa. The sampler is operated at 220 V, 50 Hz, with a built-in power supply module. The counter-rotating fan is coupled to an automotive mass air flow (MAF) sensor (KIMISS Air Flow Sensor 06A906461) which operates at 12 V and provides a 0–5 V analogue output depending on the flow rate. The MAF sensor measures the downward flow rate as the air flows through the filter into the counter rotating fan. An Arduino microcontroller controls the fan speed through PWM signals, and the analogue voltage from the MAF sensor is also measured by the Arduino microcontroller. We have used a MAF sensor as in other previous COTS ventilators designed by this group [25]. Using the calibration curve of the MAF sensor, a third-order polynomial function has been derived and is input into the Arduino microcontroller. The sampler runs a 30 s test calibration with every new filter mounted on the sampler to determine the pressure drop and automatically controls the speed of the fan to match the desired flow rate. The sampler is equipped with temperature, pressure, and relative humidity sensors that monitor the ambient conditions during sampling and logs the data onto a microSD card.

The filter holder, restraint, and components that encounter the filter are made of 3D-printed ABS plastic, which is chemically inert to alcohol used to sterilize the surface before every experiment. The housing of the sampler is made of aluminium profiles and acrylic panels that are hermetically sealed to make it weatherproof. The components used in the construction of the bioaerosol sampler, the assembled core of the sampler, the sampler core mounted inside the housing, and the interface are shown in Figure 1.

The portable bioaerosol sampler has a touch screen liquid crystal display (LCD), which has a simple and robust graphical user interface (GUI) that facilitates the operation of the sampler. The filter holder has a magnetic locking mechanism that holds the filter in place and allows quick interchangeability of filters in field minimizing contact with the filter sampling surface. Table 1 lists the specifications of the developed portable bioaerosol sampler.

The auto-compensation of pressure drop depending upon the filter and the magnetic loading lock of the filters has been designed to scale the sampler to autonomous operation with ability to change filters for unattended sampling over a long period of time.

### 2.2. Commercial Filter Selection

We narrowed down four potential filters for the experiments—polytetrafluoroethylene (PTFE), polyether sulfone (PES), polycarbonate (PC), and TissuQuartz. PTFE and PES are membrane filters made of a complex internal structure of pores within which particles are deposited as air moves through them. The PC filter is a flat filter where the particles are deposited on the surface of the filter as air moves through them. TissuQuartz is a fibrous filter made of randomly oriented fibres instead of pores, constituting a matrix. These filters have been tested for their efficiency in entrapping the bioaerosols [7,8,9,14,16,17,21,26,27]. PTFE, PES, and PC filters have a diameter of 47 mm and a pore size ranging from 0.2–0.22 µm. The TissuQuartz filter used has a diameter of 47 mm and a thickness of 432 µm. These filters are commonly used to collect airborne particles for environmental monitoring and industrial hygiene. All the filters were autoclaved at 121 °C before the experiments. The specifications of the filters used are listed in Table 2.

### 2.3. Bioaerosol Chamber

**Figure 2:**
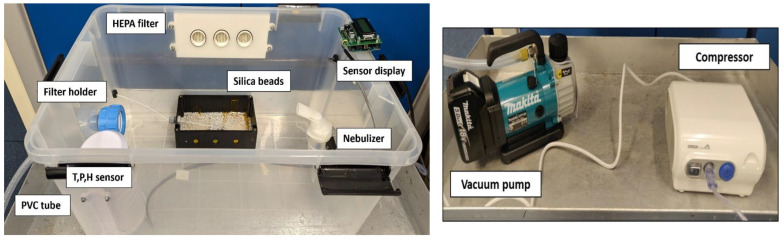
Laboratory-built portable bioaerosol chamber. This portable system consists of a filter holder that holds nucleopore filters of 47-mm diameter attached to a vacuum pump that helps bioaerosol suction. A nebulizer connected to a compressor generates the aerosols. The HEPA filter draws in clean air and stabilizes the pressure within the box.

A 50 L rectangular plastic air-tight storage box with dimensions 59 × 39 × 29 cm was used to construct the bioaerosol chamber, see Figure 2. A 47 mm polypropylene aerosol filter holder from Advantec was fixed at one of the breadths of the box using a cable gland assembly to maintain the hermetic seal of the box. The filter holder was connected to a battery-operated 18 V Makita vacuum pump using a braided 10 mm PVC tube. The vacuum pump has a flow rate of 50 L/min. A nebulizer (Omron Healthcare, C28P) of capacity 2–7 mL was used as a source for generating bioaerosols of 2.65 µm median mass aerodynamic diameter. Bioaerosol in the ambient air are composed of varied particle sizes ranging between 0–10 µm [28], with the highest concentration in the PM2.5 particle size [29]. The Omron C28P nebulizer was chosen specifically to match the droplet size produced with the highest concentration particle size of bioaerosols. The nebulizer was fixed at the opposite end of the filter holder, as shown in Figure 1, to a compressor generating 0.5 mL/min of aerosols. A HEPA filter with dimensions 21 cm × 9 cm was fixed hermetically to the box to equalize the pressure inside and outside and prevent the box from collapsing inward due to the vacuum inside. Generating bioaerosols from the nebulizer saturates the relative humidity inside the box. In order to have the relative humidity at a constant level, silica gel desiccants were placed inside the chamber. A heating pad was kept beneath the silica gel to dry the silica gel after each experiment with the lids opened. Temperature, pressure, and relative humidity were monitored in real time inside the chamber using a BME280 sensor breakout board from Adafruit. The sensor was interfaced to an Arduino UNO board and connected through USB to a laptop to record the data through TeraTerm serial data logging software in real time. 

At the beginning of every sampling, the forceps, nebulizer, and filter holder were sterilized with UV radiation for 30 min after cleaning them with Chemgene HLD_4_L (Medimark Scientific Ltd., Chester, UK). The autoclaved filter was then placed on the filter holder, and the nebulizer was primed with the bioaerosol supernatant, which was atomized to generate the bioaerosol load. The lids were then fastened, and the experiment began by running the vacuum pump for 15 min, sampling approximately 450 L of air. After each round of sampling, the chamber and the filter holder were cleaned using Chemgene HLD_4_L. 

### 2.4. Bioaerosol Supernatant and Controls

Local garden soil from Aberdeen, Scotland, was sieved, and 10 g of it was transferred to a sterile 50 mL falcon tube to which 15 mL of nuclease-free water (NEB, UK) was added and vortexed. The tubes were centrifuged at 10,000 rpm for 2–3 min, and the resultant supernatant containing the microbes was used as the liquid source for the nebulizer to generate bioaerosols. For each sampling round, 3 mL of the supernatant was freshly transferred into the nebulizer to maintain a constant volume for every fresh filter. A positive control was included to mitigate the bias in filter efficiency due to varying soil biomass with each replicate. The supernatant of constant biomass obtained after centrifuging 4 g of yeast cells from *Saccharomyces cerevisiae* (YSC-2, SigmaAldrich) dissolved in 15 mL of nuclease-free water (NEB, UK) was tested as a positive control. Nuclease-free water was used as a negative control. All the sample preparations and transfers were conducted in a laminar air flow hood. 

### 2.5. DNA Extraction

Three DNA extraction kits from Qiagen were tested, namely DNeasy PowerSoil Pro kit, DNeasy Powerlyzer PowerSoil kit, and DNeasy PowerWater kit with a few modifications. Before extraction, the addition of sonication and heat incubation at 65 °C using an ultrasonicated bath on their ambient air sampled filters led to a mean total DNA yield of more than a ten-fold increase [7]. All three Qiagen kits were tested for their DNA extraction yield on all four filters with the generated bioaerosols with positive and negative controls. Replicates of the experiment were performed with and without the sonication step. DNeasy Powerlyzer Powersoil kit was the most efficient in extracting quantitative DNA from the sampled filters post-sonication at 45 KHz. The extraction protocol was optimized as follows: 

**Step 1**: The sampled filters were cylindrically rolled and aseptically transferred into the Powerlyzer glass bead tubes provided in the kit, to which 1 mL of Powerbead solution and 80 µL of C1 lysis buffer were added. The filters can also be cut into small pieces using sterile scissors.

**Step 2**: The tubes were then placed in the sonicator, operating at 45 KHz at 65 °C for 30 min. 

**Step 3**: Post sonication, the powerbead tubes were secured to a bead beater (BeadBug™ Microtube homogeniser) and homogenized at 3500 RPM for 30 s. 

**Step 4**: Hereafter, the extraction procedure follows the manufacturer’s instructions, including an additional n washing step using C5 wash solution. To minimise DNA loss during the extraction procedure, pipetting as much volume of supernatant after the lysis step is essential. Using a 100–200 µL tip helps maximum recovery of the supernatant due to its thin tapered end and ease of fitting inside the tube. The volume of all the solutions provided in the kit at each step is recalculated based on the supernatant obtained with each filter.

**Step 5**: The final DNA is eluted in 30 µL of nuclease-free water and quantified using Qubit^®^ 4.0 fluorometer (Thermo Fisher Scientific^®^, Paisley, UK) as well as NanoDrop^®^ One spectrophotometer (Thermo Fisher Scientific^®^, Paisley, UK). 

**Figure 3:**
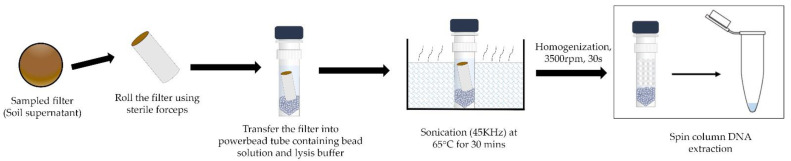
Summary of the optimised DNA extraction protocol from steps 1–4. The sonication of the sampled filter in a warm bath and bead beating are the key steps in the extraction process as they significantly improve the extraction efficiency.

The summary of the extraction protocol is elucidated in the Figure 3 as follows: 

## 3. Results

### 3.1. Bioaerosol Chamber

The efficiency of each filter was tested with a slightly varying biomass for the nebulized soil supernatant sampling, while the tests for yeast sampling were performed with constant biomass as a positive control. Nuclease-free water was also sampled as a negative control for each filter type to avoid the inclusion of any false positives in either of the sampling rounds. After 15 min of sampling each round, the efficiency of the filters was assessed. 

The sensors, which logged the temperature and pressure parameter, noted an average value of 20–22 °C and 1000–1005 mbar for all filter types. By contrast, relative humidity significantly varied with the filter type and the bioaerosol source. The environmental conditions inside the chamber at the time of the experiment are detailed in Table 3. With soil, yeast, and water as aerosol sources, the highest humidity was recorded to be 83%, 78%, and 40%, respectively. Humidity levels predominantly impact filter efficiency and microbial viability, which in turn also depends on the structure of the microbial cell. For example, lipid (20–30%) and non-lipid enveloped viruses tend to survive in lower and higher humidity (70–90%) levels, respectively. For bacteria, various factors, including cell shape, wall structure, and means of respiration, influence their viability in humid conditions [30].

**Figure 4:**
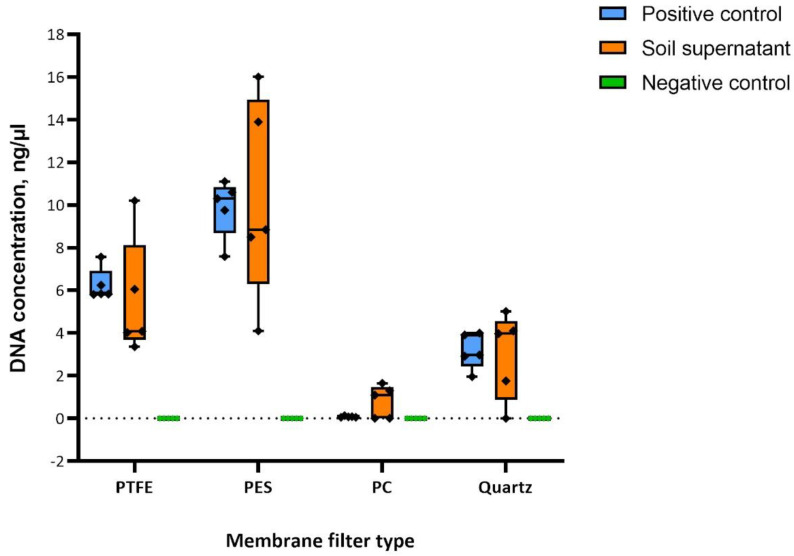
Results of bioaerosol chamber sampling experiments. The boxplot represents five biological replicates (*n* = 5) for each membrane filter type. Yeast cells were used as a positive control whose mass was constant with each replicate, and varying biomass of soil supernatant was used to validate the consistency of the filter efficiency. The graph was plotted using Prism 9. Error bars indicate the mean with standard deviation.

The order of efficiency was found to be PES, PTFE, TissuQuartz, and PC, with PES being the most efficient and PC being the least, as shown in the boxplot in Figure 4. Qubit^®^ 4.0 Fluorometer with Qubit^®^ dsDNA HS (High Sensitivity) Assay Kit has been used in the quantitative analysis of the yielded DNA owing to its specificity at low DNA concentrations of 10 pg/mL [31]. PES and PTFE showed few similar extraction efficiencies concerning soil supernatant. With yeast samples, PES was consistent among the four. Negative controls showed no quantifiable DNA, negating the possibility of cross-contamination from the laboratory, chamber environment, or kit reagents. 

### 3.2. Outdoor Environment

Based on the results obtained from the bioaerosol chamber experiments, the bioaerosol sampling and DNA extraction methodology were validated in the representative outdoor environment using PES and PTFE membrane filters. The environmental and meteorological conditions on the sampling days have not been considered variables in this study. 

**Figure 5:**
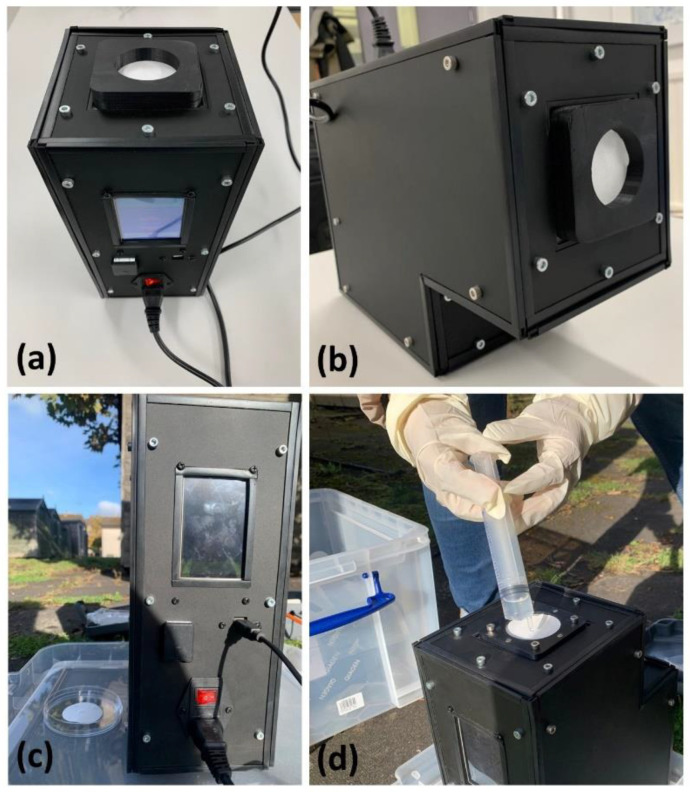
(**a**,**b**) Bioaerosol sampler loaded with the 47 mm diameter filter; (**c**) sampling in an outdoor environment; and (**d**) 1X PBS added to moisten the filter.

The sampler was wiped using 70% isopropyl alcohol spray cleaner (RS components, UK) and set up in an open backyard as shown in Figure 5, wherein a sterile PES/PTFE filter was aseptically fitted into the 47 mm filter holder and run for 24 h at 150 L/min. Previous studies have shown that a long sampling time can lead to desiccation of the microbes, which in turn affects the stability of the DNA. For example, Gram-negative bacteria have very low tolerance toward desiccation, and 24 h of exposure to those cells while sampling can lead to loss of genetic material [32]. A few drops of sterile 1X PBS buffer were added to the filter using a sterile syringe before sampling, just enough to keep the filter moist and to mitigate desiccation. As controls, the DNA extraction procedure was followed for (1) a filter fitted to a passive sampler, which collects bioaerosols via gravity, and (2) an unopened sterile filter that negates any false DNA quantification due to kit reagents or laboratory contamination. Both the controls had consistently no sign of DNA that could be quantified by Qubit^®^ 4.0 fluorometer. While PTFE filters provided no quantifiable amount of DNA, a considerable amount of genetic material was extracted from PES filters. From three tests, each run for 24 h at 150 L/min, a total of 4.14 ng, 4.44 ng, and 2.07 ng of DNA was extracted, which were quantified using 1X HS DNA kit of Qubit^®^ 4.0 fluorometer. 

### 3.3. Post-Sampling Filter Micro-Scale Characterization

**Figure 6:**
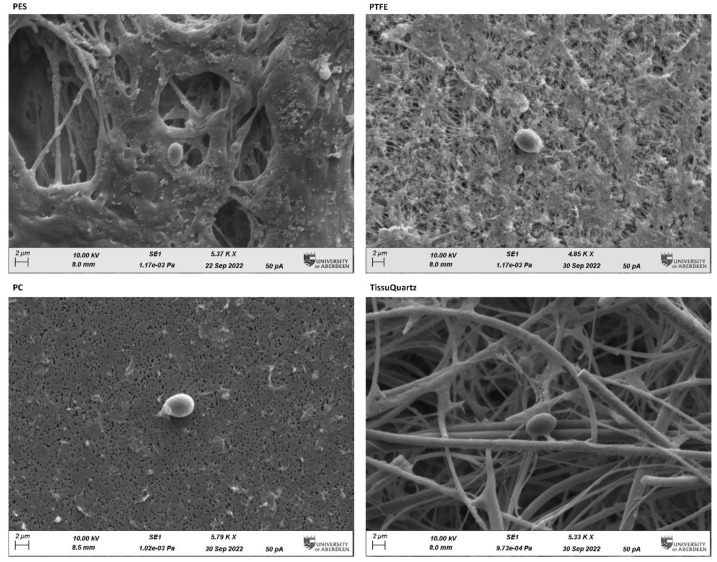
SEM images captured at ~5.00 K X Zoom using 10.00 KV electron beam on the post-sampled filters subjected to yeast bioaerosol generated within the constrained chamber. The oval-shaped single yeast cell can be distinctively seen captured onto the different filters. The microscale characterization of the filters using SEM imaging reveals the morphology and structure of the filters and their influence on trapping bioaerosol particles. PES (hydrophilic) and PTFE (hydrophobic) have good loading capacity due to higher surface area of entrapment. PC is a flat filter and has the least surface area for bioaerosol loading. TissueQuartz is a fibrous filter and has good loading capacity but suffers from lower efficiency compared to membrane filters.

The PTFE filter performed quite closely to the PES filter in the constrained environment of the bioaerosol chamber. To illustrate how the morphology of the filter and its physicochemical properties affect the sampling and extraction efficiency, we performed scanning electron microscopy (SEM) of all four filters after sampling with bioaerosol positive controls in the bioaerosol chamber. The SEM images were captured at the Microscopy and Histology Core Facility at the University of Aberdeen. Figure 6 shows the SEM images of the filters with the yeast cell captured onto the filter media. SEM images show clearly visible micro-scale morphological differences among the four filters. In all images, single yeast cells are clearly distinguished over the background of the filters. PES and PTFE are membrane filters with characteristic complex pore structures, PC being a flat filter observable as a 2D plane and TissuQuartz showing the fibrous structure. 

## 4. Discussion

The experiments have narrowed down PES as an optimal filter for bioaerosol sampling to recover quality DNA using the standardized DNA extraction protocol. The significant amount of DNA obtained from the sampling in the outdoor environment can be further amplified for either 16S bacteria, viruses, or fungi based on preferred taxonomic profiling and sequenced using next-generation sequencing (NGS) technologies. 

Our study suggests that membrane and fibrous filters offer more surface area for bioaerosol collection due to their complex morphology. This can be observed in the results shown in Figure 6, where PC has the lowest DNA yield compared to the other three filters. With the low microbial load in the air, increasing the collection surface area is critical to improving the filter’s loading capacity, and membrane and fibrous filters offer more surface area per square mm of the filter. Though membrane filters are like fibrous filters in entrapping bioaerosol particles, they have shown good performance compared to fibrous filters for bioaerosol collection in the literature [3]. Fibrous filters have a poor loading capacity compared to membrane filters for the same filter thickness. The filtration process in the fibrous filter takes place through the whole filter width, and its efficiency increases with this thickness, which in contrast to the membrane filter, and remains unaffected by the thickness of the filter as the filtration takes place mainly on the surface of the filter [33]. This can also be accounted for from the filter specifications listed in Table 2. The high flow rates of TissuQuartz allow most of the microbes to pass through, affecting its loading capacity.

PES and PTFE are very similar in specifications and mainly vary in their physicochemical properties. Bacteria, except for a few extremophiles, are generally hydrophilic [34]. Pollen grains across various plant species have a microcapsule structure with the outermost layer of sporopollenin, a strong, crosslinked biopolymer made of lipids and a pollen kit [35]. They are amphiphilic and adhere to polar liquids, similar to yeast [36], which explains the similar performance of PES and PTFE in the bioaerosol chamber. The more hydrophilic cells adhere more strongly to hydrophilic surfaces and vice versa [37,38]. Most microbes have a hydrophilic exterior, so they adhere well to hydrophilic surfaces. PES is hydrophilic, and this physicochemical difference from PTFE allows PES to have a better bioaerosol loading capacity, resulting in a higher DNA yield.

## 5. Conclusions

We have compared the four most used filters in aerobiology and concluded that the PES membrane filter is the optimal filter for bioaerosol sampling for genetic material extraction with promising results of up to 4 ng of DNA from bioaerosols captured over 24 h at a sampling rate of 150 L/min. After filtration in a controlled environment, SEM images of the filters clearly show the single yeast cells and their attachment to the filter. In this study, we have demonstrated the successful use of a new optimized, portable, closed bioaerosol sampler based on membrane filters built using commercial off-the-shelf components. This sampler can operate autonomously outdoors for prolonged times, capturing ambient bioaerosols and avoiding user contamination. We first performed a comparative analysis in a controlled environment to select the optimal active membrane filter based on its ability to capture and extract DNA. A bioaerosol chamber was designed for this purpose, and three commercial DNA extraction kits were tested. Our analysis shows no contamination in the control tests and optimal extraction with a 0.22 µm polyether sulfone (PES) membrane filter. The bioaerosol sampler was tested outdoors in a representative environment and run for 24 h at 150 L/min. Our analysis suggests that this method can recover up to 4 ng of DNA within one day of ambient monitorization. This is sufficient to be used for genomic applications. This system, along with the robust extraction protocol, can be automated for continuous environmental monitoring to gain insights into the time evolution of microbial communities within the air. This will provide continuous air quality monitoring for a wide range of applications, from agriculture, waste management, occupational health, and medicine to defense.

## Figures and Tables

**Table 1:** Specifications of the bioaerosol sampler.

Parameter	Range
Voltage	220 V AC 50 Hz
Power	50 W
Filter size	47 mm diameter
Sampling time	30 min–72 h
Sampling flow rate	50–250 L/min (intervals of 50 L/min)
Temperature sensor characteristics	−40 °C to +85 °C; accuracy: ±1 °C; resolution: 0.010 °C
Humidity sensor characteristics	5% to 95% RH; accuracy: ±3%; resolution: 0.7%
Pressure sensor characteristics	300–1100 mbar; accuracy: ±2 mbar; resolution: 0.06 mbar

**Table 2:** Specifications of the filter used in this study.

Filter	Product ID	Hydrophobic/Hydrophilic	Thickness	Air Flow Rate
PES	GPWP04700	Hydrophilic	160–185 µm	6 L/min × cm^2^
PTFE	FGLP04700	Hydrophobic	150 µm	5 L/min × cm^2^
PC	GTTP04700	Hydrophilic	7–22 µm	0.007 L/min × cm^2^
TissuQuartz	513-0028	Hydrophilic	432 µm	73 L/min × cm^2^

**Table 3:** Environmental parameters during the experiments inside the bioaerosol chamber.

Filter Type	Aerosol Source	Average T (°C)	Highest RH%	Average P (Bar)
	Yeast supernatant	20.91	61.78	1.002
PTFE	Soil supernatant	20.86	65.92	1.005
	Negative control	22.40	38.72	1.002
	Yeast supernatant	21.34	75.57	1.003
PES	Soil supernatant	20.82	70.72	1.005
	Negative control	22.57	40.02	1.002
	Yeast supernatant	21.68	83.68	1.003
PC	Soil supernatant	20.68	79.39	1.009
	Negative control	22.30	45.53	1.002
	Yeast supernatant	21.52	53.02	1.002
Quartz	Soil supernatant	21.12	61.39	1.005
	Negative control	22.24	39.42	1.003
